# High-dose aciclovir in CMV infection prophylaxis after allogeneic HSCT: a single-center long-term experience

**DOI:** 10.1038/s41409-023-02081-6

**Published:** 2023-08-23

**Authors:** Tomáš Kabut, Barbora Weinbergerová, František Folber, Martina Lengerová, Jiří Mayer

**Affiliations:** 1 Department of Internal Medicine—Hematology and Oncology, University Hospital Brno, Brno, Czech Republic; 2 Department of Internal Medicine—Hematology and Oncology, Faculty of Medicine, Masaryk University, Brno, Czech Republic

**Keywords:** Disease prevention, Therapeutics

## Abstract

There is only limited data on cytomegalovirus (CMV) prophylaxis with high-dose (HD) aciclovir after allogeneic hematopoietic stem cell transplantation (allo-HSCT). We performed a retrospective analysis on a total of 179 patients who underwent their allo-HSCT with HD-aciclovir prophylaxis at our center. A clinically significant CMV infection (cs-CMVi) was observed in 56 (31%) cases with a median time of 49 (range 25–147) days after HSCT. A significantly higher CMV infection rate was observed in seropositive recipients with a seronegative donor (74%) compared to seropositive recipients with a seropositive donor, and seronegative recipients with seropositive and seronegative donors (24%, 18%, 7% respectively; *p* < 0.001). The CMV serostatus was the only significant risk factor for CMV infection in our analysis. CMV disease developed in three patients with CMV-related death in two cases. During HD-aciclovir prophylaxis, we did not observe any medical condition attributable to HD-aciclovir’s adverse effects. Compared to published results, we observed a low incidence of cs-CMVi with HD-aciclovir prophylaxis in several patient subgroups, especially in seropositive recipients with a seropositive donor. With respect to the determined threshold, HD-aciclovir prophylaxis seems to have good efficacy in an intermediate cs-CMVi risk patients, but prospective randomized trials would be needed for definite conclusions.

## Introduction

CMV is the most clinically important viral infection in patients after allogeneic hematopoietic stem cell transplantation and is associated with great morbidity and mortality [1, 2]. Frequent CMV viremia monitoring and early preemptive antiviral therapy is the most utilized strategy to prevent progression to end-organ disease in many transplant centers [3, 4]. With this preemptive approach, the incidence of CMV disease is reduced to less than 10% [5]. Although the CMV disease incidence remains low, CMV reactivation with subsequent preemptive treatment is associated with a significant increase in non-relapse mortality [1]. Myelosuppression after antiviral treatment with secondary bacterial and fungal infections or increased risk of graft versus host disease in patients with CMV reactivation are the most important causes of worse overall outcome [2, 6]. Effective and safe anti-CMV prophylaxis could reduce the risk of CMV reactivation and improve mortality after HSCT. Several virostatic agents such as ganciclovir and foscarnet have proven effective in reducing the risk of CMV infection and disease, but significant organ toxicity is a major limitation in its usage as prophylaxis [2, 7–10]. Primary prophylaxis with letermovir led to a reduction in cs-CMVi with a favorable toxicity profile [11], and is recommended for CMV prophylaxis after allo-HSCT in CMV seropositive recipients (CMV R+) [3, 4]. Nevertheless, in some countries and transplant centers, the limited availability or financial burden of letermovir may limit its use in daily practice. Before letermovir’s introduction, several other antiviral drugs were evaluated for the prevention of CMV infection and disease. Besides other CMV effective drugs such as ganciclovir or foscarnet, data about high-dose (HD) aciclovir use in CMV prophylaxis had previously been published [10, 12–19]. Some of these analyses showed the HD-aciclovir’s efficacy in prophylaxis of CMV disease and reactivation. Based on these results, the primary CMV prophylaxis after allo-HSCT with HD-aciclovir has been routinely used for more than 20 years in our center. Our retrospective, single-center analysis presents the results of long-term experience with HD-aciclovir for CMV prophylaxis after transplantation in allo-HSCT recipients.

## Methods

### Patients

We performed a retrospective analysis of 179 consecutive allo-HSCT recipients transplanted at our institution between 2015 and 2019 with CMV HD-aciclovir prophylaxis. All patients who received HD-aciclovir for at least 1 day were included in the analysis, no other inclusion or exclusion criteria were used. The time period of 5 years between 2015 and 2019 was selected with the aim of ensuring consistency in terms of factors such as pre-transplantation characteristics, type of preparation regime, GvHD prophylaxis or the quality of supportive care and meets the current standards in real clinical practice and enable better comparison to recent data. Analysis was performed in accordance with local law and with the approval of the local ethics committee. Informed consent was obtained from all patients.

### HD-aciclovir administration, conditioning regimen and GvHD prophylaxis

Since day −2 of transplantation, HD-aciclovir was administered intravenously in doses of 500 mg/m^2^ three times a day or 800 mg four times a day orally. Prophylaxis was given for 6 months in CMV seronegative recipients (CMV R−) and 12 months in seropositive recipients. In all patients, triple graft versus host disease (GvHD) prophylaxis with pretransplant in vivo antithymocyte globulin (ATG Grafalon) T-lymphodepletion (total dose 30 mg/kg for related and 60 mg/kg for unrelated donors) was administered. Combined with ATG, cyclosporine A (3 mg/kg daily from day −1) and methotrexate (15 mg/m^2^ day 1 and 10 mg/m^2^ day 3, 6 and 11) or mycophenolic acid (15 mg/kg twice a day from the day of transplant) was used. Conditioning regimens used in our cohort are shown in Table 1.

**Table 1:** Patient characteristics.

	*n* = 179 (%)
Sex—male	102 (57)
Age—median (min–max)	47.6 (18.4–66.2) years
Diagnosis	
Acute leukemia	107 (60)
CLL/PLL/HCL	17 (9)
CML	6 (3)
MDS/MPN	25 (14)
NHL/HL	21 (12)
AA	3 (2)
Donor type	
MSD	45 (25)
10/10	94 (52)
9/10	39 (22)
HAPLO	1 (1)
Conditioning intensity	
MAC	35 (20)
10 Gy TBI/Cy	16 (9)
CyBu	17 (9)
FluBu4	2 (1)
RIC	144 (80)
FLAMSA + 4 Gy TBI/Cy	120 (67)
8 Gy TBI/Flu	4 (2)
FluBu2	15 (8)
Other	5 (3)
CMV serostatus	
R+	127 (71)
R+/D+	87 (49)
R+/D−	40 (22)
R−	52 (29)
R−/D−	30 (17)
R−/D+	22 (12)

Patient characteristics according to diagnosis, donor type, conditioning intensity and CMV serostatus.

*CLL* chronic lymphocytic leukemia, *PLL* prolymphocytic leukemia, *HCL* hairy cell leukemia, *CML* chronic myeloid leukemia, *MDS* myelodysplastic syndrome, *MPN* myeloproliferative neoplasms, *NHL* non-Hodgkin’s lymphomas, *HL* Hodgkin’s lymphoma, *AA* aplastic anemia, *MSD* matched sibling donor, *MUD* matched unrelated donor, *HAPLO* haploidentical donor, *MAC* myeloablative conditioning regimens, *RIC* reduced intensity conditioning regimens, *CMV* cytomegalovirus, *R* recipient, *D* donor, *TBI* total body irradiation, *Cy* cyclophosphamide, *Bu* busulfan, *Flu* fludarabine, *FLAMSA* fludarabine + Ara-C + amsacrine.

### CMV surveillance and management

CMV-specific whole blood quantitative real-time PCR (polymerase chain reaction) to monitor CMV was performed weekly during hospitalization and then at every medical visit. Before May 2017, CMV surveillance was performed with validated “in house” CMV DNA real-time PCR assay (copies/ml). After May 2017, CMV monitoring was performed using the Biomérieux “CMV R-Gene” real-time PCR kit (IU/ml). A viremia higher than 2500 IU/ml (or 500 copies/µg DNA) was considered a clinically significant CMV reactivation/infection (cs-CMVi) leading to the initiation of preemptive anti-CMV treatment. The threshold values used at our center to initiate preemptive treatment are based on our historical experience and analyses, in which a spontaneous decrease was mostly observed without need of preemptive treatment in patients whose viral loads did not reach the limit of 2500 IU/ml (500 copies/µg PCR respectively).

### Statistical analysis

Basic statistical methods were used to describe absolute and relative frequency for categorical variables, and mean, median and ranges for continuous variables. Fisher’s exact test was used to evaluate categorical parameters and Mann–Whitney *U* test for continuous variable correlations. All reported *p* values are two-sided and *p* values < 0.05 were considered statistically significant.

## Results

### Baseline characteristics

A total number of 179 consecutive patients who underwent their allo-HSCT between 2015 and 2019 at our center were included in the analysis. There were slightly more men than women (102; 57%), the median age was 47.6 years (range 18.4–66.2 years) and median follow-up was 827 days after allo-HSCT (range 1–2325 days). As hematopoietic stem cells donors, most frequently matched unrelated donors (10 out of 10) were used in more than half of the patients (94; 53%), matched sibling donors in one-quarter of patients (45; 24%), followed by partially matched unrelated donor (9 out of 10) in the rest of the patients (39; 22%), respectively. A haploidentical transplant was performed in one patient only (1; 1%). Reduced-intensity conditioning (RIC) was used in 144 (80%) patients and myeloablative conditioning (MAC) in only 35 (20%) patients. The most common indications for allo-HSCT were acute leukemias (107; 60%), followed by myelodysplastic syndrome or myeloproliferative neoplasms (25; 14%), non-Hodgkin and Hodgkin lymphomas (21; 12%), chronic lymphocytic leukemia or prolymphocytic leukemia or hairy cell leukemia (17; 9%), chronic myeloid leukemia (6; 3%) and aplastic anemia (3; 2%), respectively. According to CMV serostatus, the most common combination of recipient and donor (recipient/donor CMV serostatus) was positive/positive (87; 49%) followed by positive/negative (40; 22%), negative/negative (30; 17%) and negative/positive (22; 12%). Baseline characteristics are described in Table 1.

### CMV reactivation

**Fig. 1:**
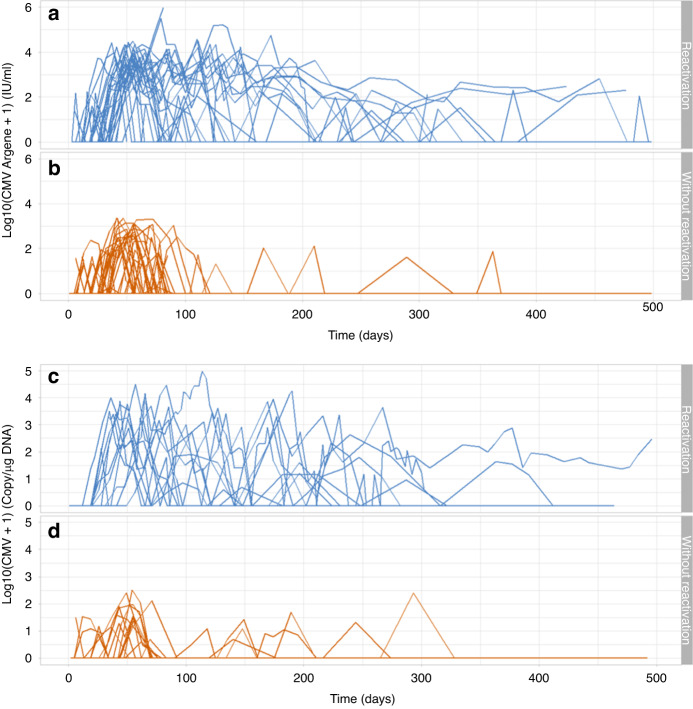
Kinetics of CMV viral loads. Logarithmic values of CMV viral loads in all patients from day of transplantation to day 500 post-transplant according to presence of cs-CMVi and used method. **a** CMV viral loads in patients with cs-CMVi (IU/ml); **b** CMV viral loads in patients without cs-CMVi (IU/ml); **c** CMV viral loads in patients with cs-CMVi (copy/µg DNA); **d** CMV viral loads in patients without cs-CMVi (copy/µg DNA).

Out of a total 179 patients, cs-CMVi occurred in 56 (31%) cases. Detailed data on CMV reactivation are summarized in Table 2 and data on CMV viral load kinetics for patients with and without cs-CMVi are shown in Fig. 1. Median time to cs-CMVi was 49 days (range 25–147 days) with 96% of cs-CMVi within the first 100 days after HSCT. In the CMV seropositive recipients, the cs-CMV reactivation was seen in 50 of 127 (39%) patients, compared to 6 of 52 (12%) patients in CMV seronegative recipients (*p* < 0.05). Considering donor CMV serostatus, the differences between subgroups were even more pronounced. The most frequent cs-CMVi was observed in CMV seropositive recipients with seronegative donors, compared to all other combinations (29 of 40 vs. 27 of 139; 73% vs. 19%; *p* < 0.001). The frequency of cs-CMVi in other combinations of recipients and donors (R/D) were 21 of 87 (24%) in CMV R+/D+, 4 of 22 (18%) in CMV R−/D+ and two of 30 (7%) in CMV R−/D−, respectively. The difference in cs-CMVi rate was statistically significant in CMV R+/D− group compared to all other subgroups (*p* < 0.001 for all three subgroups). In contrast to these results, the differences were not statistically significant between CMV R+/D+ and CMV R−/D+ (*p* = 0.777) and with a borderline significance in CMV R−/D− (*p* = 0.059) patients. Median time to cs-CMVi did not differ significantly between the high-risk group CMV R+/D− and others (47 vs. 55 days; *p* = 0.066). Regarding the HLA status, cs-CMVi was less frequent in patients with matched related or unrelated donors than patients with an HLA mismatch, however, this difference was not statistically significant (41 of 139 vs. 15 of 40; 30% vs. 38%; *p* = 0.340). Taking both potential risk factors together, cs-CMVi was present in 12 (50%) cases in patients with both risk factors (CMV R+ and HLA mismatch), in 41 (35%) patients with only one risk factor (HLA mismatch or CMV R+) and 3 (8%) patients with no risk factor. Cs-CMVi was most common among patients with acute leukemias (37 of 107; 35%), myelodysplastic, and myeloproliferative diseases including CML (11 of 31; 36%). In lymphoproliferative diseases (without acute lymphoblastic leukemia), cs-CMV reactivation was less common (8 of 38; 21%). The difference between these two groups was also not statistically significant (*p* = 0.168).

**Table 2:** CMV reactivation.

Time to CMV reactivation—median (min–max)	49 (25–147) days
CMV viremia—median (min–max)^a^	
All patients (all results^b^)	394 (range 1–978,083) IU/ml
	73 (range 1–97,400) copies/µg DNA
With cs-CMVi (all results^b^)	486 (range 6–978,083) IU/ml
	112 (range 1–97,400) copies/µg DNA
With cs-CMVi (at the time of diagnosis)	5819.5 (range 2619–157,343) IU/ml
	2379 (range 514–47,756) copies/µg DNA
Without cs-CMVi (all results)	105 (range 1–2325) IU/ml
	27 (range 1–1637) copies/µg DNA
	*N* (%)
Total patients with cs-CMVi reactivation	56/179 (31)
CMV serostatus	
R+	50/127 (39)
R+/D+	21/87 (24)
R+/D−	29/40 (74)
R−	6/52 (12)
R−/D−	2/30 (7)
R−/D+	4/22 (18)
Donor type	
MSD + MUD 10 out of 10	41/139 (30)
9 out of 10 + HAPLO	15/40 (38)
CMV serostatus and HLA combinations	
CMV R+ and HLA mismatch	12/24 (50)
CMV R+ or HLA mismatch	41/119 (35)
CMV R− and without HLA mismatch	3/36 (8)
Conditioning intensity	
MAC	9/35 (26)
RIC	47/144 (33)
Diagnosis	
Acute leukemia	37/107 (35)
CLL/PLL/HCL	3/17 (18)
CML	2/6 (33)
MDS/MPN	9/25 (36)
NHL/HL	5/21 (24)
AA	0/3 (0)

Data on CMV viral loads and CMV reactivation occurrence in whole patient group and according to CMV serostatus, donor type, conditioning intensity and diagnosis.

*CLL* chronic lymphocytic leukemia, *PLL* prolymphocytic leukemia, *HCL* hairy cell leukemia, *CML* chronic myeloid leukemia, *MDS* myelodysplastic syndrome, *MPN* myeloproliferative neoplasms, *NHL* non-Hodgkin’s lymphomas, *HL* Hodgkin’s lymphoma, *AA* aplastic anemia, *MSD* matched sibling donor, *MUD* matched unrelated donor, *HAPLO* haploidentical donor, *MAC* myeloablative conditioning regimens, *RIC* reduced intensity conditioning regimens, *CMV* cytomegalovirus, *cs-CMVi* clinically significant CMV infection, *R* recipient, *D* donor, *HLA* human leukocyte antigen.

^a^Negative results excluded.

^b^Counted from all results, including the values before cs-CMVi development.

### CMV disease

The progression to CMV disease was observed in three cases with CMV-related death in two cases (three of 179 patients; 2%). Diagnoses of these patients were chronic lymphocytic leukemia, primary myelofibrosis, and chronic myeloid leukemia in blast crisis. CMV disease manifested as a gastrointestinal (GIT) disease, meningoencephalitis or pneumonia. CMV end-organ disease was verified by immunohistochemistry (GIT) or by PCR positivity from cerebrospinal or bronchoalveolar lavage fluid. All three patients received reduced-intensity conditioning (FLAMSA + RIC TBI4/Cy or FluBu2). In a patient with CML blast crisis, an unplanned switch to a haploidentical transplant with post-transplant cyclophosphamide (+ cyclosporine A and ATG immunosuppression) had to be performed due to an infection of the scheduled matched donor after starting the conditioning regimen. The other two patients had matched unrelated donors (9 out of 10 and 10 out of 10). In all three patients, the first cs-CMVi developed within 100 days after HSCT (71, 48, and 76 days), but a subsequent CMV disease occurred much later (576 and 193 days after HSCT) after several courses of CMV preemptive treatment for recurrent cs-CMVi in two cases. Both patients were treated for chronic graft versus host disease (GvHD) at the same time. The third patient (haplo-HSCT) developed a fulminant refractory CMV disease 78 days after HSCT with fast progression to respiratory failure and death, despite intensive antiviral treatment and supportive care including extracorporeal membrane oxygenation. Summary is shown in Table 3.

**Table 3:** CMV disease.

Patient number	1	2	3
Sex	Male	Female	Male
Age	68 years	59 years	58 years
Diagnosis	CLL	PMF	CML blast crisis
Conditioning	FluBu2	FLAMSA + RIC TBI4/Cy	FLAMSA + RIC TBI4/Cy
Donor type	MUD 10 out of 10	MUD 9 out of 10	HAPLO
CMV serostatus	R+/D+	R+/D−	R−/D+
GvHD (grade 3–4)	GIT	Skin	None
CMV disease	Colitis	Encephalitis	Pneumonia
Time from HSCT to first CMV reactivation	71 days	48 days	76 days
Time from HSCT to CMV disease	576 days	193 days	78 days
Outcome	Remission	Death due to CMV	Death due to CMV

Data on patients with CMV disease—patient and transplant characteristics, graft versus host disease presence, CMV disease type and outcome.

*CMV* cytomegalovirus, *CLL* chronic lymphocytic leukemia, *PMF* primary myelofibrosis, *CML* chronic myeloid leukemia, *MUD* matched unrelated donor, *HAPLO* haploidentical donor, *R* recipient, *D* donor, *GvHD* graft versus host disease, *GIT* gastrointestinal tract, *HSCT* hematopoietic stem cell transplantation.

### Graft versus host disease

In the whole patient cohort, acute GvHD (aGvHD) was observed in 63 (35%) patients in a median of 48 days (range 11–100 days) after allo-HSCT. Regarding the presence of cs-CMVi, the aGvHD developed more often in patients with cs-CMVi than patients without (25 of 56 vs. 38 of 123; 45% vs. 31%), but the difference did not reach the statistical significance (*p* = 0.0916). Median time to aGvHD development in patients with cs-CMVi was 51 days (range 21–100 days) after transplantation. In 68% of these cases, GvHD preceded the cs-CMVi development with median time of 10 days (range 0–92 days) from aGvHD to cs-CMVi development. In both groups (with vs. without cs-CMVi), most of the aGvHD cases were grade I–II (23 of 25 vs. 33 of 38; 92% vs. 87%) with only few cases of severe GvHD grade III–IV (2 of 25 vs. 5 of 38; 8% vs. 13%). In general, patients with aGvHD grade II–III were treated with systemic corticosteroids (methylprednisolone 1–2 mg/kg or equivalent) as a first line treatment, while patients with mild aGvHD grade I were treated with systemic corticosteroids or topical corticosteroids only as an alternative considering the affected organ, general conditions, and other comorbidities. Data regarding chronic GvHD (cGvHD) were available for 173 patients, in whom cGvHD was observed in 32 (18%) cases with a median of 130 days (range 100–490 days) post-transplant. In 25 of 32 (75%) cases cGvHD was preceded by the presence of aGvHD. Similar to aGvHD, cGvHD was present more often in patients with cs-CMVi than patients without (15 of 56 vs. 17 of 123; 28% vs. 14%) with a difference at the limit of statistical significance (*p* = 0.056).

### EBV and other viral reactivations

Together with CMV viremia monitoring, the EBV viremia in blood was also monitored at the same time. The indication for preemptive rituximab administration was repeated EBV viremia above 80,000 IU/ml (or 1000 copies/μg DNA), in patients with significant viremia under this cut-off level, reduction of immunosuppressive therapy and intensive monitoring was indicated. In patients with any EBV viremia level present, the median viral load was 1413 IU/ml (range 4–2,260,748 IU/ml) or 86.5 copies/μg DNA respectively (range 1–2,060,220 copies/μg DNA). The threshold for significant EBV viremia was reached in 39 (22%) patients with median viral load 3405 IU/ml (range 4–2260 748 IU/ml) or 232 copies/μg DNA (range 4–5946 copies/μg DNA) respectively (all results included, including the values before clinically significant EBV reactivation development and after its treatment). However, the preemptive treatment with rituximab was administered in only 14 (8%) patients with repeated significant positivity in the confirmatory sample, or extremely high viral load. The median viral load for these subgroup of patients was 5675 IU/ml (range 58–2,260,748 IU/ml) or 384 copies/μg DNA (range 6–5946 copies/μg DNA) respectively (all results included, including the values before clinically significant EBV reactivation development and after its treatment). Among patients with significant EBV reactivation treated with rituximab, cs-CMVi developed in 7 of 14 (50%) cases. Other viral infections (adenoviruses (AdV), herpes simplex virus (HSV), human herpesvirus 6 (HHV-6), varicella zoster virus) are not routinely monitored at our center and are assessed only in cases of clinical suspicion or as part of a broad differential diagnosis. In our patient cohort, the presence of viremia in two or more consecutives samples was observed in five patients. All these cases were HHV-6 viremia, and tests for chromosomally integrated HHV-6 was not performed. Two patients with repeated HHV-6 positivity and clinical suspicion for reactivation/infection were treated with ganciclovir. No AdV, VZV or systemic HSV reactivation cases (presence of viremia) were diagnosed in our patient cohort.

### Mortality

All-cause mortality in our cohort during the entire follow-up was 41% (74 patients), with a median time from HSCT to death of 150.5 days (range from day −1 to 1870 days after HSCT). All-cause mortality at day 100, 180 and 365 after HSCT was 17%, 22% and 30%, respectively. Overall, the cause of death was related to relapse of the disease in 40% of cases. In the rest of the patients (60% of cases), infections, liver sinusoidal obstruction syndrome/veno-occlusive disease (SOS/VOD), and acute GvHD were the most common causes of death. As mentioned previously, only two deaths were directly attributed to CMV infection/disease (2 of 74; 3%). The mortality was higher in patients who did not experience cs-CMV reactivation than the cs-CMV reactivation group in all three analyzed time-points (22% vs. 5% at day 100; 28% vs. 7% at day 180, and 34% vs. 20% at day 365). These differences were statistically significant at day 100 and 180 (*p* = 0.005 and *p* = 0.002) and had a borderline significance at day 365 (*p* = 0.054). According to recipient CMV serostatus, 55 of 127 (43%) patients died in the seropositive group and 19 of 52 (37%) in the seronegative group (*p* = 0.504). When evaluating the mortality in the CMV R+/D− (20 of 40; 50%) group to other CMV serostatus combinations (54 of 139; 39%), the difference was also not significant (*p* = 0.274), although we observed a far lower mortality rate in the CMV seronegative recipient group and CMV R/D combinations other than R−/D+.

### Toxicity

The toxicity of HD-aciclovir prophylaxis in our cohort was not assessed. Due to the retrospective character of the study with the absence of a control group, we were not able to indicate the relationship between common adverse events such as renal insufficiency or liver function test elevations with HD-aciclovir use. These events are most likely related to direct conditioning toxicity, infections, SOS/VOD, GvHD, and others. In the context of common myelosuppression after CMV-active antiviral agents such as ganciclovir and valganciclovir, we evaluated the time to engraftment after HSCT in our cohort. In the whole cohort the median time to engraftment was 20 days (range 10–40 days) for neutrophils and 17 days (range 5–379 days) for platelets, according to the EBMT (European Group for Blood & Marrow Transplantation) definition (sustained neutrophil count higher than 0.5 × 10^9^/l and platelets >20 × 10^9^/l with no transfusion support, respectively) [20]. In patients with cs-CMVi and subsequent ganciclovir/valganciclovir preemptive treatment, there was no difference in time to neutrophil recovery compared to patients without cs-CMVi (median 20 (range 11–40) vs. 20 (range 10–39) days; *p* = 0.490), nor in time to platelets recovery (median 18 (range 7–75) vs. 16 (range 5–379) days; *p* = 0.535). The difference was not observed even in the case of neutrophil recovery above 1 × 10^9^/l (median 26 (range 14–55) vs. 24 (range 10–145) days; *p* = 0.689). In general, HD-aciclovir prophylaxis was well tolerated. We did not observe any medical condition attributable to HD-aciclovir’s adverse effects nor any HD-aciclovir prophylaxis interruption due to toxicity.

## Discussion

We retrospectively evaluated our long-term experience with the use of HD-aciclovir in CMV prophylaxis after allo-HSCT, which resulted in a low incidence of clinically significant CMV infection in several patient groups. High-dose aciclovir is commonly used in herpes simplex and varicella zoster infections prophylaxis, where it is efficient even in low doses. However, there are only scarce data on its effectiveness in the prophylaxis of CMV disease and reactivation/infection. In the first analyses in the 1980s and 1990s, aciclovir was compared to a placebo for CMV disease prophylaxis [12–14]. The authors of these analyses presented a decrease in the CMV disease risk with an improvement in overall survival, but the length of administration and aciclovir doses varied. Currently, with routine monitoring of viremia/antigenemia and early preemptive treatment, CMV disease develops in only 1–2% of patients, and advanced CMV disease prophylaxis is no longer considered a sufficient goal [2, 3]. Above that, the CMV disease diagnosis in these analyses is based on virus isolation or serology [12–14] and does not meet the current standards in CMV diagnosis. Later, several non-randomized trials, one non-randomized study with historical-controls comparison and two randomized studies (aciclovir vs. valaciclovir/ganciclovir) evaluated the CMV reactivation risk in various groups of patients after allo-HSCT [10, 15–19]. In the first randomized trial, the HD-aciclovir prophylaxis was compared to ganciclovir in 91 seropositive allo-HSCT recipients. Prophylaxis was administered from engraftment until day 100 post-transplant and patients were monitored with CMV antigenemia once weekly with preemptive therapy in case of positivity. Although lower cumulative incidence of CMV antigenemia was observed in the ganciclovir group than the HD-aciclovir group (31% vs. 41%; *p* = 0.22), the difference didn’t reach statistical significance, partially due to low patient numbers. The incidence of CMV disease in the study was generally higher, but comparable between both groups (13% vs. 17%; *p* = 0.59). In conclusion, the authors did not find a statistically significant difference between ganciclovir and aciclovir when used as part of an overall strategy to prevent CMV reactivation and disease in allo-HSCT, although fewer side-effects occurred with aciclovir prophylaxis [10]. In the second randomized trial HD-aciclovir prophylaxis vs. valaciclovir prophylaxis was evaluated in 727 CMV seropositive or seronegative allo-HSCT recipients, both drugs administered from day 28 until week 18 post-transplant. Although there was a trend for some differences according to R/D CMV serostatus, CMV infection was generally less common in the valaciclovir group than the HD-aciclovir group (28% vs. 40%; *p*  < 0.0001), with no difference in CMV disease incidence and survival between groups [18]. It should be noted that in both studies mentioned, HD-aciclovir was used during the pre-engraftment period in all patients. Among other published analyses, a non-randomized study of 43 seropositive umbilical cord blood transplant recipients evaluated the efficacy of the „intensive strategy“ of CMV prevention with HD-aciclovir or valaciclovir compared to 29 historical controls with standard low-dose aciclovir prophylaxis. Prophylaxis with HD-aciclovir/valaciclovir was administered until day 100 post-transplant in combination with frequent CMV viremia monitoring twice a week (any presence of viremia detected from serum by PCR was considered as clinically significant). In this high-risk population, the cumulative incidence of CMV reactivation was a significantly lower in “intensive strategy” group than the control group (60% vs. 100%; *p* < 0.001) [19]. In the other non-randomized studies, clinically significant CMV viremia or antigenemia occurred in a wide range of 26–88% of patients with HD-aciclovir prophylaxis, according to patient risk and the cut-offs used for significant reactivation. Due to the inconsistency of these results, there is still no clear conclusion about HD-aciclovir CMV prophylaxis’ effectiveness, as well as, for example, the influence of various risk factors on the effectiveness of prophylaxis.

In general terms, which are similar to our results, the recipient CMV seropositivity is considered the most significant risk factor for CMV reactivation after HSCT. Clinically significant CMV reactivation is reported in up to 80% of seropositive patients after HSCT with a standard preemptive approach, and the highest risk in combination with a seronegative donor [3, 21–23]. The major impact on the recipient and donor CMV serostatus was also observed in our cohort, in which the CMV R+/D− subgroup developed CMV reactivation in 74% of cases. In these patients, HD-aciclovir did not lead to a reduction in cs-CMVi risk compared to the published results on a standard approach without CMV prophylaxis. On the other hand, in the CMV R+/D+ subgroup, we observed a significant reduction to only 24% of patients in cs-CMVi incidence compared to 30–50% cs-CMVi incidence in CMV R+/D+ according to previous reports with large numbers of patients [1, 22, 24–26]. It is noteworthy that the ATG T-lymphodepletion routinely used in all our patients was administered in only a limited group of patients (in about 30% or less) in most of the analyses [1, 22, 24, 26]. T-lymphodepletion is often considered an additional risk factor for cs-CMVi with a reported cs-CMVs incidence of about 50% in the CMV R+/D+ subgroup [27], in contrast to our experience with only 24% of cs-CMVs in CMVR+/D+ patients with ATG lymphodepletion. These results suggest that HD-acyclovir appears to have good efficacy in preventing cs-CMVi in CMV R+/D+ patients and may therefore represent a suitable option for CMV prophylaxis for this subgroup of patients.

Currently, the only drug recommended for CMV prophylaxis is the terminase-complex inhibitor letermovir [3, 4]. In the registration randomized trial, a significant reduction in cs-CMVi in CMV seropositive recipients occurred in 17.5% of patients by week 24 compared to 41.8% in the placebo arm [11]. Some later real-world data reported a higher proportion of cs-CMVi with an increasing incidence of cs-CMV reactivation after day 100 in patients with letermovir prophylaxis. Herein the cs-CMVi reached 20.0–43.2% [28–31] compared to 39–59% reactivation rate in historic control groups (both seronegative and seropositive recipients included) [28, 30, 31]. In our cohort, the cs-CMVi rate in seropositive recipients was 39% with the previously mentioned significant difference according to donor serostatus. Specific data according to donor serostatus in letermovir analyses are mostly unavailable, but there is no evidence of different effectiveness regarding donor serostatus. Although we did not observe the HD-aciclovir efficacy in CMV R+/D− patients, cs-CMV reactivation incidence in CMV R+/D+ did not differ from letermovir prophylaxis in a real-world setting (24% vs. 20.0–43.2%, respectively). Interestingly, 96% cs-CMVi in our analysis developed before day 100 as breakthrough infections in high-risk patients. During letermovir prophylaxis, a low cs-CMV reactivation rate was observed (7.7%) with the increased cs-CMV rate in the post-prophylactic period to 17.5% by week 24 [11]. A possible explanation may be the delayed maturation of anti-CMV T-cell immunity caused by reduced antigenic exposure through letermovir administration, as suggested by certain recent reports [32, 33]. Subclinical reactivations during HD-aciclovir prophylaxis may potentially lead to sufficient antigenic stimulation, which is associated with earlier specific immunity reconstitution [34, 35] and still preserves adequate efficacy in cs-CMVi prevention in the intermediate risk group (CMV R+/D+).

Our study is not entitled to make a definite conclusion about the effectiveness of HD-acyclovir in the CMV reactivation prophylaxis due to certain limitations, especially its retrospective nature with no control group. Also the limitation for the comparison with published data may be a higher viremia threshold for cs-CMVi used at our institution (2500 IU/ml or 500 copies/µg DNA). However, even with the viremia threshold used, the median CMV viral load in patients without cs-CMVi was significantly lower than this threshold (105 IU/ml or 27 copies/µg DNA) and even in case of hypothetical reassessing the group of patients without cs-CMVi to a lower threshold for cs-CMVi (1000 IU/ml or 150 copies/µg DNA), the majority of these patients still did not meet these adjusted criteria.

Regarding the mortality data, due to the absence of a control group in our analysis, we are not able to make any conclusion about the impact of HD-aciclovir prophylaxis on mortality outcomes. Also in agreement with published mortality data, we assume the major influence of variables such as pretransplant characteristics including the hematological malignancy type, conditioning regimen intensity, ATG use, and others. Overall all-cause mortality in our cohort on day 100 (17%) and 365 (29%) were higher than large recently published EBMT analysis (≈10.1% on day 100; ≈23.2% on day 365) [36]. Evaluating the mortality outcome in particular subgroups, we noted significantly higher mortality up to day 180 after HSCT in patients without cs-CMVi (28% vs. 7%, *p* = 0.002). No significant difference in mortality was found either between seropositive and seronegative recipients (43% vs. 37%, *p* = 0.504), or between R+/D− and other R/D serocombinations (50% vs. 39%, *p* = 0.274), although a trend in favor of seronegative recipients and combinations other than R+/D− was observed. These results do not correspond to published data, where CMV reactivation and CMV seropositivity in the recipient itself are associated with higher treatment related mortality and overall mortality [1, 24, 37] The probable explanation for this difference is most likely the relatively low number of patients for the mortality assessment in our study, especially in the patient subgroups with cs-CMVi and CMV R+/D− patients.

## Conclusion

In conclusion, with HD-aciclovir use in cs-CMVi prophylaxis after allo-HSCT, we observed low cs-CMVi incidence in a significant number of patients compared to previously published data. The significance of our results is limited by the retrospective nature of the analysis with the absence of a control group. However, it describes the long-term experience of one institution in a relatively homogeneous specific patient group after a hematopoietic stem cell transplant with in vivo T-lymphodepletion. In our cohort, the most clinically significant results were seen in CMV seropositive recipients with a seropositive donor, in whom the risk of cs-CMVi during HD-aciclovir prophylaxis was significantly reduced compared to published results. At the same time, CMV-specific immunity reconstitution probably wasn’t negatively affected, which is important to prevent late CMV reactivations. Regarding our results, HD-aciclovir seems to be an option for the prophylaxis of CMV reactivation in intermediate risk patients (CMV R+/D+, T-lymphodepletion etc.), for example in cases of letermovir unavailability or financial obstacles, or in low-risk CMV seronegative recipients. To validate these results, as well as those previously published, a prospective, randomized and well-designed trial with high-dose aciclovir would be necessary. To prevent CMV disease, a combination of HD-aciclovir prophylaxis and a standard preemptive approach (CMV viremia monitoring and early preemptive treatment) is required. Among the highest-risk CMV R+/D− patients, we did not observe sufficient efficacy of HD-aciclovir and in these patients, letermovir plays an irreplaceable role.

## Data Availability

The datasets generated during and/or analyzed during the current study are available from the corresponding author on reasonable request.

## References

[1] Teira P, Battiwalla M, Ramanathan M, Barrett AJ, Ahn KW, Chen M (2016). Early cytomegalovirus reactivation remains associated with increased transplant-related mortality in the current era: a CIBMTR analysis. Blood..

[2] Chen K, Cheng MP, Hammond SP, Einsele H, Marty FM (2018). Antiviral prophylaxis for cytomegalovirus infection in allogeneic hematopoietic cell transplantation. Blood Adv.

[3] Ljungman P, de la Camara R, Robin C, Crocchiolo R, Einsele H, Hill JA (2019). Guidelines for the management of cytomegalovirus infection in patients with haematological malignancies and after stem cell transplantation from the 2017 European Conference on Infections in Leukaemia (ECIL 7). Lancet Infect Dis.

[4] Hakki M, Aitken SL, Danziger-Isakov L, Michaels MG, Carpenter PA, Chemaly RF (2021). American Society for Transplantation and Cellular Therapy Series: #3-Prevention of cytomegalovirus infection and disease after hematopoietic cell transplantation. Transpl Cell Ther.

[5] Erard V, Guthrie KA, Seo S, Smith J, Huang M, Chien J (2015). Reduced mortality of cytomegalovirus pneumonia after hematopoietic cell transplantation due to antiviral therapy and changes in transplantation practices. Clin Infect Dis.

[6] Cantoni N, Hirsch HH, Khanna N, Gerull S, Buser A, Bucher C (2010). Evidence for a bidirectional relationship between cytomegalovirus replication and acute graft-versus-host disease. Biol Blood Marrow Transplant.

[7] Reed DR, Petroni GR, West M, Jones C, Alfaraj A, Williams PG (2023). Prophylactic pretransplant ganciclovir to reduce cytomegalovirus infection after hematopoietic stem cell transplantation. Hematol Oncol Stem Cell Ther.

[8] Bacigalupo A, Tedone E, Van Lint MT, Trespi G, Lonngren M, Sanna MA (1994). CMV prophylaxis with foscarnet in allogeneic bone marrow transplant recipients at high risk of developing CMV infections. Bone Marrow Transplant.

[9] Bregante S, Bertilson S, Tedone E, Van Lint MT, Trespi G, Mordini N (2000). Foscarnet prophylaxis of cytomegalovirus infections in patients undergoing allogeneic bone marrow transplantation (BMT): a dose-finding study. Bone Marrow Transplant.

[10] Burns LJ, Miller W, Kandaswamy C, DeFor TE, MacMillan ML, Van Burik JA (2002). Randomized clinical trial of ganciclovir vs acyclovir for prevention of cytomegalovirus antigenemia after allogeneic transplantation. Bone Marrow Transplant.

[11] Marty FM, Ljungman P, Chemaly RF, Maertens J, Dadwal SS, Duarte RF (2017). Letermovir prophylaxis for cytomegalovirus in hematopoietic-cell transplantation. N Engl J Med.

[12] Gluckman E, Lotsberg J, Devergie A, Zhao XM, Melo R, Gomez-Morales M (1983). Prophylaxis of herpes infections after bone-marrow transplantation by oral acyclovir. Lancet..

[13] Meyers JD, Reed EC, Shepp DH, Thornquist M, Dandliker PS, Vicary CA (1988). Acyclovir for prevention of cytomegalovirus infection and disease after allogeneic marrow transplantation. N Engl J Med.

[14] Prentice HG, Gluckman E, Powles RL, Ljungman P, Milpied N, Fernandez Rañada JM (1994). Impact of long-term acyclovir on cytomegalovirus infection and survival after allogeneic bone marrow transplantation. European Acyclovir for CMV Prophylaxis Study Group. Lancet.

[15] Hazar V, Kansoy S, Küpesiz A, Aksoylar S, Kantar M, Yeşilipek A (2004). High-dose acyclovir and pre-emptive ganciclovir in prevention of cytomegalovirus disease in pediatric patients following peripheral blood stem cell transplantation. Bone Marrow Transplant.

[16] Hazar V, Ugur A, Colak D, Saba R, Tezcan G, Kupesiz A (2006). Cytomegalovirus antigenemia and outcomes of patients undergoing allogeneic peripheral blood stem cell transplantation: effects of long-term high-dose acyclovir prophylaxis and preemptive ganciclovir treatment. Jpn J Infect Dis.

[17] Nakamura R, Cortez K, Solomon S, Battiwalla M, Gill VJ, Hensel N (2002). High-dose acyclovir and pre-emptive ganciclovir to prevent cytomegalovirus disease in myeloablative and non-myeloablative allogeneic stem cell transplantation. Bone Marrow Transplant.

[18] Ljungman P, de La Camara R, Milpied N, Volin L, Russell CA, Crisp A (2002). Valacyclovir International Bone Marrow Transplant Study Group. Randomized study of valacyclovir as prophylaxis against cytomegalovirus reactivation in recipients of allogeneic bone marrow transplants. Blood.

[19] Milano F, Pergam SA, Xie H, Leisenring WM, Gutman JA, Riffkin I (2011). Intensive strategy to prevent CMV disease in seropositive umbilical cord blood transplant recipients. Blood..

[20] Valcárcel D, Sureda A. Graft failure. In: Carreras E, Dufour C, Mohty M, Kröger N, editors. The EBMT handbook: hematopoietic stem cell transplantation and cellular therapies. Chapter 41, 7th ed. Cham (CH): Springer; 2019. 32091673

[21] Ljungman P, Hakki M, Boeckh M (2011). Cytomegalovirus in hematopoietic stem cell transplant recipients. Hematol Oncol Clin North Am.

[22] George B, Pati N, Gilroy N, Ratnamohan M, Huang G, Kerridge I (2010). Pre-transplant cytomegalovirus (CMV) serostatus remains the most important determinant of CMV reactivation after allogeneic hematopoietic stem cell transplantation in the era of surveillance and preemptive therapy. Transpl Infect Dis.

[23] Einsele H, Ljungman P, Boeckh M (2020). How I treat CMV reactivation after allogeneic hematopoietic stem cell transplantation. Blood.

[24] Schmidt-Hieber M, Labopin M, Beelen D, Volin L, Ehninger G, Finke J (2013). CMV serostatus still has an important prognostic impact in de novo acute leukemia patients after allogeneic stem cell transplantation: a report from the Acute Leukemia Working Party of EBMT. Blood.

[25] Zhou W, Longmate J, Lacey SF, Palmer JM, Gallez-Hawkins G, Thao L (2009). Impact of donor CMV status on viral infection and reconstitution of multifunction CMV-specific T cells in CMV-positive transplant recipients. Blood..

[26] Takenaka K, Nishida T, Asano-Mori Y, Oshima K, Ohashi K, Mori T (2015). Cytomegalovirus reactivation after allogeneic hematopoietic stem cell transplantation is associated with a reduced risk of relapse in patients with acute myeloid leukemia who survived to day 100 after transplantation: The Japan Society for Hematopoietic Cell Transplantation Transplantation-related Complication Working Group. Biol Blood Marrow Transplant.

[27] Turki AT, Tsachakis-Mück N, Leserer S, Crivello P, Liebregts T, Betke L (2022). Impact of CMV reactivation on relapse of acute myeloid leukemia after HCT is dependent on disease stage and ATG. Blood Adv.

[28] Liu LW, Yn A, Gao F, Olson M, Crain M, Abboud R (2022). Letermovir discontinuation at day 100 after allogeneic stem cell transplant is associated with increased CMV-related mortality. Transpl Cell Ther.

[29] Anderson A, Raja M, Vazquez N, Morris M, Komanduri K, Camargo J (2020). Clinical "real-world" experience with letermovir for prevention of cytomegalovirus infection in allogeneic hematopoietic cell transplant recipients. Clin Transplant.

[30] Hiraishi I, Ueno R, Watanabe A, Maekawa S (2021). Safety and effectiveness of letermovir in allogenic hematopoietic stem cell transplantation recipients: interim report of post-marketing surveillance in Japan. Clin Drug Investig.

[31] Yoshimura H, Satake A, Ishii Y, Ichikawa J, Saito R, Konishi A (2022). Real-world efficacy of letermovir prophylaxis for cytomegalovirus infection after allogeneic hematopoietic stem cell transplantation: a single-center retrospective analysis. J Infect Chemother.

[32] Zamora D, Duke ER, Xie H, Edmison BC, Akoto B, Kiener R (2021). Cytomegalovirus-specific T-cell reconstitution following letermovir prophylaxis after hematopoietic cell transplantation. Blood.

[33] Marzolini MAV, Mehra V, Thomson KJ, Tholouli E, Bloor AJC, Parker A (2021). Letermovir prophylaxis in T-cell-depleted transplants: breakthrough and rebound infections in the postmarketing setting. Blood Adv.

[34] Blyth E, Withers B, Clancy L, Gottlieb D (2016). CMV-specific immune reconstitution following allogeneic stem cell transplantation. Virulence.

[35] Hakki M, Riddell SR, Storek J, Carter RA, Stevens-Ayers T, Sudour P (2003). Immune reconstitution to cytomegalovirus after allogeneic hematopoietic stem cell transplantation: impact of host factors, drug therapy, and subclinical reactivation. Blood.

[36] Styczyński J, Tridello G, Koster L, Iacobelli S, van Biezen A, van der Werf S (2020). Infectious Diseases Working Party EBMT. Death after hematopoietic stem cell transplantation: changes over calendar year time, infections and associated factors. Bone Marrow Transplant.

[37] Giménez E, Torres I, Albert E, Piñana JL, Hernández-Boluda JC, Solano C (2019). Cytomegalovirus (CMV) infection and risk of mortality in allogeneic hematopoietic stem cell transplantation (Allo-HSCT): a systematic review, meta-analysis, and meta-regression analysis. Am J Transplant.

